# Homologous or heterogenous vaccination boosters enhance neutralizing activities against SARS‐CoV‐2 Omicron BA.1 variant

**DOI:** 10.1002/mco2.143

**Published:** 2022-05-11

**Authors:** Zhongcheng Zhou, Peng Du, Ning Li, Xinxin Xiong, Shengjun Tang, Qinjin Dai, Taorui Wang, Meixing Yu, Miao Man, Kelvin Lam, Daniel T. Baptista‐Hon, Wa Hou Tai, Olivia Monteiro, Weng Sam Ng, Un Man Lee, Zhihai Liu, Kang Zhang, Gen Li

**Affiliations:** ^1^ Guangzhou Women and Children's Medical Center Guangzhou Medical University Guangzhou P. R. China; ^2^ University Hospital and Center for Biomedicine and Innovations, Faculty of Medicine Macau University of Science and Technology Macau P. R. China; ^3^ St. Januario Central Hospital Macau P. R. China; ^4^ Department of Bioinformatics and AI Guangzhou Laboratory Guangzhou P. R. China

**Keywords:** booster, neutralization, Omicron BA.1, SARS‐CoV‐2, vaccine

## Abstract

The SARS‐CoV‐2 Omicron BA.1 variant of concern contains more than 30 mutations in the spike protein, with half of these mutations localized in the receptor‐binding domain (RBD). Emerging evidence suggests that these large number of mutations impact the neutralizing efficacy of vaccines and monoclonal antibodies. We investigated the relative contributions of spike protein and RBD mutations in Omicron BA.1 variants on infectivity, cell–cell fusion, and their sensitivity to neutralization by monoclonal antibodies or vaccinated sera from individuals who received homologous (CoronaVac, SinoPharm) or heterologous (CoronaVac—BNT162b2, BioNTech) and nonhuman primates that received a recombinant RBD protein vaccine. Our data overall reveal that the mutations in the spike protein reduced infectivity and cell–cell fusion compared to the D614G variant. The impaired infectivity and cell–cell fusion were dependent on non‐RBD mutations. We also find reduced sensitivity to neutralization by monoclonal antibodies and vaccinated sera. However, our results also show that nonhuman primates receiving a recombinant RBD protein vaccine show substantial neutralization activity. Our study sheds light on the molecular differences in neutralizing antibody escape by the Omicron BA.1 variant, and highlights the promise of recombinant RBD vaccines in neutralizing the threat posed by the Omicron BA.1 variant.

## Graphical abstract

Sera from vaccine recipients of the regimen shown was evaluated for neutralizing activity against Omicron BA.1. All sera showed impaired neutralizing activity against the Omicron BA.1. However, our results show that the efficacy of a heterologous booster regimen (two doses of CoronaVac followed by the BNT162b2 vaccine) elicited stronger neutralizing activity than a homologous booster regimen (three doses of the CoronaVac vaccine) against both D614G and Omicron BA.1 pseudoviruses.

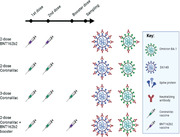

## 1 INTRODUCTION

Emerging variants of SARS‐CoV‐2 continue to modify the trajectory of the coronavirus disease 2019 (COVID‐19) pandemic.^1^, ^2^, ^3^, ^4^ The designated variant of concern, B.1.1.529 or Omicron BA.1 variant, contains over 55 mutations in the viral genome.^5^ The majority of these (over 30 mutations) are concentrated in the Spike protein, and 15 of these mutations in the receptor‐binding domain (RBD), which is the locus of viral interaction with human angiotensin converting enzyme 2 (ACE2) and subsequent entry into human cells.

A key determinant of altering the trajectory of the COVID‐19 pandemic is vaccination. A number of vaccines have been approved for emergency use. Among these, the CoronaVac vaccine (from Sinovac Biotech Ltd., China) and the BNT162b2 vaccine (from Pfizer‐BioNTech) are two of the most commonly administered vaccines around the world. The CoronaVac uses an inactivated SARS‐CoV‐2 technology, while the BNT162b2 is an mRNA vaccine. Both of these vaccines have demonstrated good efficacy in clinical trials.^6^, ^7^ However, vaccine efficacy changes with the emergence of new variants. The standard two‐dose regimens of most authorized vaccines are highly effective against previous variants of concern such as the B.1.351 (Beta variant) and B.1.617.2 (Delta variant). However, not only does vaccine effectiveness wane over time,^8^, ^9^, ^10^ the impacts of the emergence of the Beta and Delta variants have also shown changes in their sensitivity to the activity of neutralizing antibodies.^11^, ^12^ Indeed, the vaccine efficacy of the standard two‐dose regimen is low against the Omicron variant.^13^ However, booster (third) doses of vaccines can increase the level of protection against COVID‐19 when Omicron is the dominant variant.^14^, ^15^ It may do so by increasing the titer of neutralizing antibodies.^16^, ^17^, ^18^, ^19^

The unusually large number of mutations found on the spike protein may mediate the enhanced ability of the Omicron variant for immune escape and also higher transmissibility.^20^, ^21^ For example, deletion mutations, such as Δ69‐70 and Δ143‐145, in the *N*‐terminal domain of the spike protein may enhance infectivity.^21^ However, the role of the other mutations in immune escape remains to be elucidated. In this study, we determined how these nonsynonymous substitutions in the Omicron BA.1 variant affected the infectivity, the cell–cell fusion, and the neutralization activity of serum from vaccinated nonhuman primates as well as human recipients of different homologous and heterologous booster regimens.

## 2 RESULTS

### 2.1 Omicron BA.1 variants show RBD‐dependent reduction in infectivity

**FIGURE 1:**
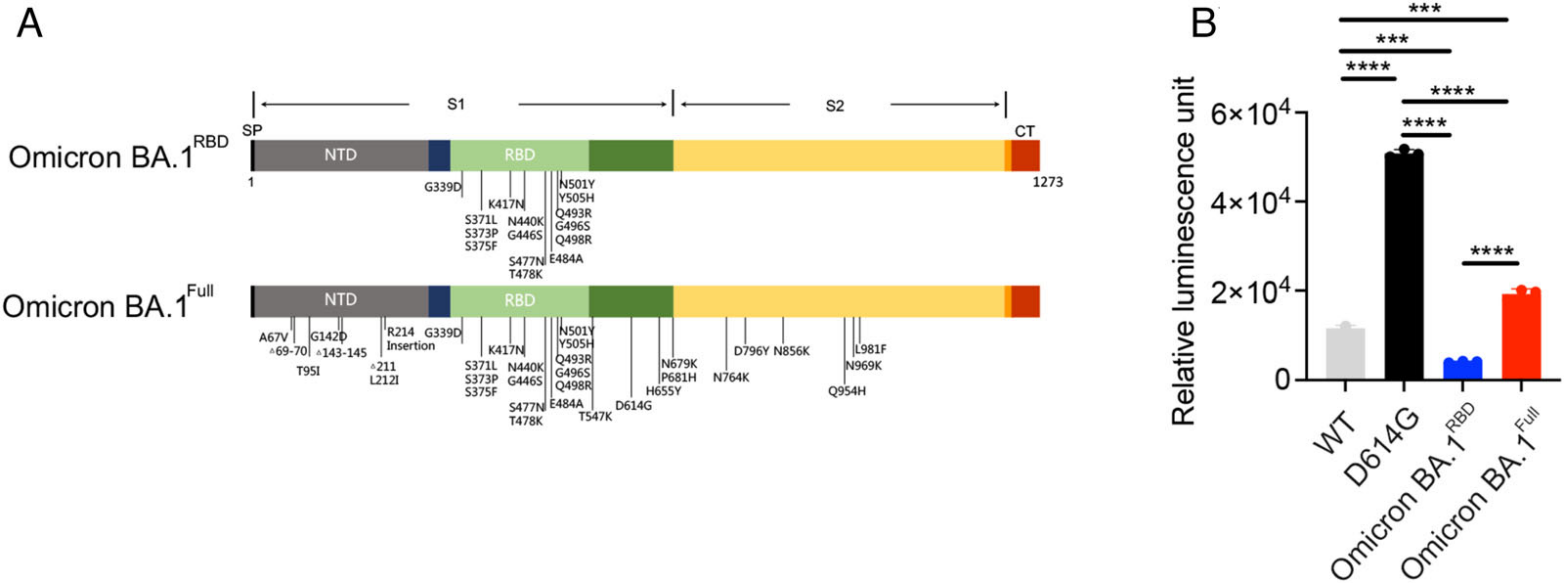
Omicron BA.1 variant spike protein mutations and infectivity. (A) Schematic diagram of the mutations found in the spike protein of the Omicron BA.1 variant. Based on these mutations, we produced pseudotyped viruses which contained the entire complement of spike protein mutations (Omicron BA.1^Full^), or only mutations in the RBD (Omicron BA.1^RBD^). (B) Mean luminescence measured from HEK‐293T cells stably expressing ACE2 and TMPRSS2 following infection with pseudotyped viruses. One‐way ANOVA analysis reveals a statistically significant increase in luminescence following infection by pseudotyped viruses containing the D614G mutation, versus all of the other variants tested. Cells infected with the Omicron BA.1^Full^ also showed higher luminescence than those infected with the Omicron BA.1^RBD^, or the WT pseudovirus

The spike protein RBD interacts with the host ACE2 to gain entry. We determined if mutations in the RBD alone are responsible for the enhanced infectivity of the Omicron BA.1 variant. We constructed two Omicron BA.1 pseudovirus containing genes encoding luciferase and eGFP, one with the full complement of mutations in the spike protein (Omicron BA.1^Full^), and the other with mutations only in the RBD (Omicron BA.1^RBD^; Figure 1A). HEK‐293T cells stably expressing ACE2 and TMPRSS2 were infected with the Omicron BA.1 pseudovirus and we measured luciferase activity to determine infectivity. Consistent with previous observations, the D614G substitution conferred enhanced infectivity versus the wild‐type (WT) SARS‐CoV‐2 (Figure 1B). The Omicron BA.1^Full^ was 1.7‐fold more infectious than WT. Interestingly, Omicron BA.1^RBD^ had a 2.8‐fold reduction in infectivity versus WT. Pseudovirus harboring the D614G substitution alone had a much higher infectivity than either the Omicron BA.1^Full^ (2.6‐fold) or the Omicron BA.1^RBD^ (12.4‐fold). Our data indicate mutations outside of the RBD play a critical role in the infectivity of the Omicron BA.1 variant.

### 2.2 Cell–cell fusion of the SARS‐CoV‐2 pseudotyped virus with the Omicron BA.1

**FIGURE 2:**
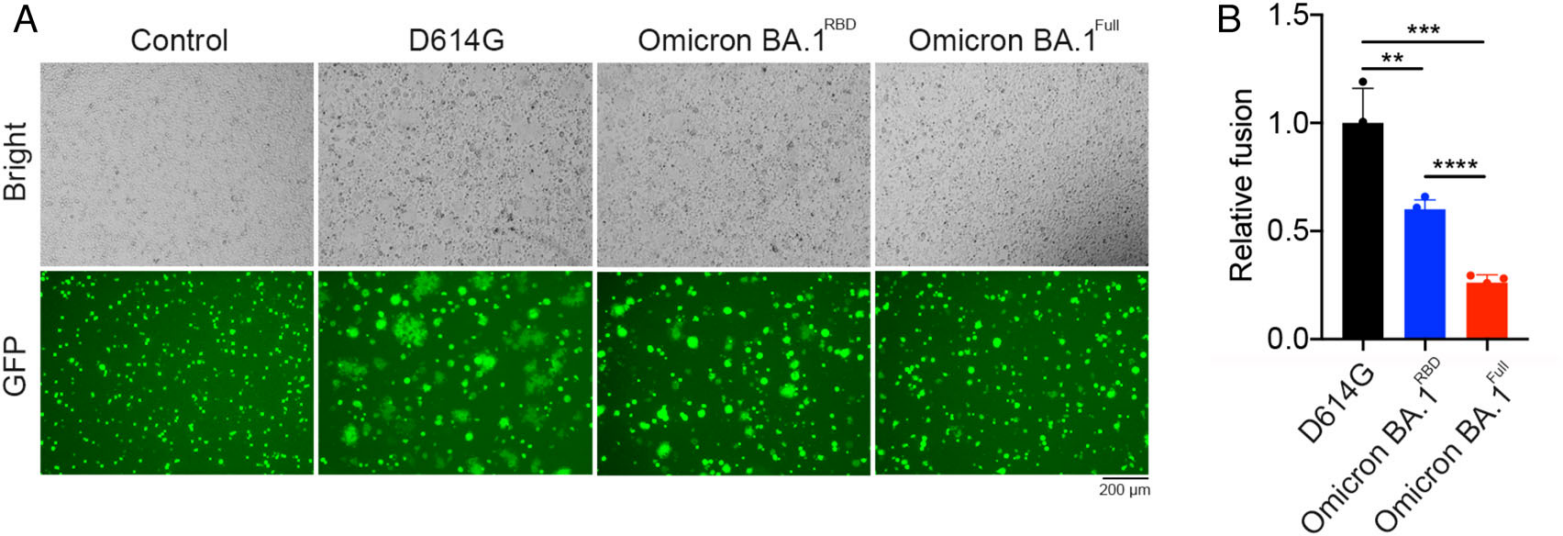
Omicron BA.1 variant spike mutations decreased cell–cell fusion. (A) Representative brightfield and fluorescence images of EGFP and spike protein transfected HEK‐293T cells cocultured with HEK‐293T cells stably expressing ACE2 and TMPRSS2. Large syncytia formation with reduced GFP intensity can be observed in cells expressing D614G, Omicron BA.1^Full^, and Omicron BA.1^RBD^ spike proteins. (B) Syncytia formation is expressed as relative fusion and mean values are plotted. Spike protein harboring the D614G mutation induced a larger extent of cell–cell fusion than the Omicron BA.1 variant spike proteins (one‐way ANOVA, *p* < 0.001). The Omicron BA.1^RBD^ spike protein also induced significantly more cell–cell fusion than the Omicron BA.1^Full^ spike protein (one‐way ANOVA, *p* < 0.0001)

SARS‐CoV‐2 may spread from cell to cell through syncytia formation resulting from cell–cell fusion.^22^ We determined the ability of Omicron BA.1^Full^ and Omicron BA.1^RBD^ to induce cell–cell fusion. We evaluated the spread of eGFP fluorescence between infected HEK‐293T cells as a measure of cell–cell fusion (Figure 2A). The numbers of fused and unfused cells were counted and expressed as a relative fusion ratio. Our data indicate that there was a reduction in the number of fused cells mediated by Omicron BA.1^Full^ or Omicron BA.1^RBD^ pseudovirus compared to the D614G pseudovirus (Figure 2B). Qualitatively, the fused cells also appeared smaller in the presence of the Omicron BA.1^Full^ or Omicron BA.1^RBD^ pseudovirus (Figure 2A). This suggests that each syncytium is formed from fewer cells.

### 2.3 Neutralization activity of Omicron BA.1 by antibodies

**FIGURE 3:**
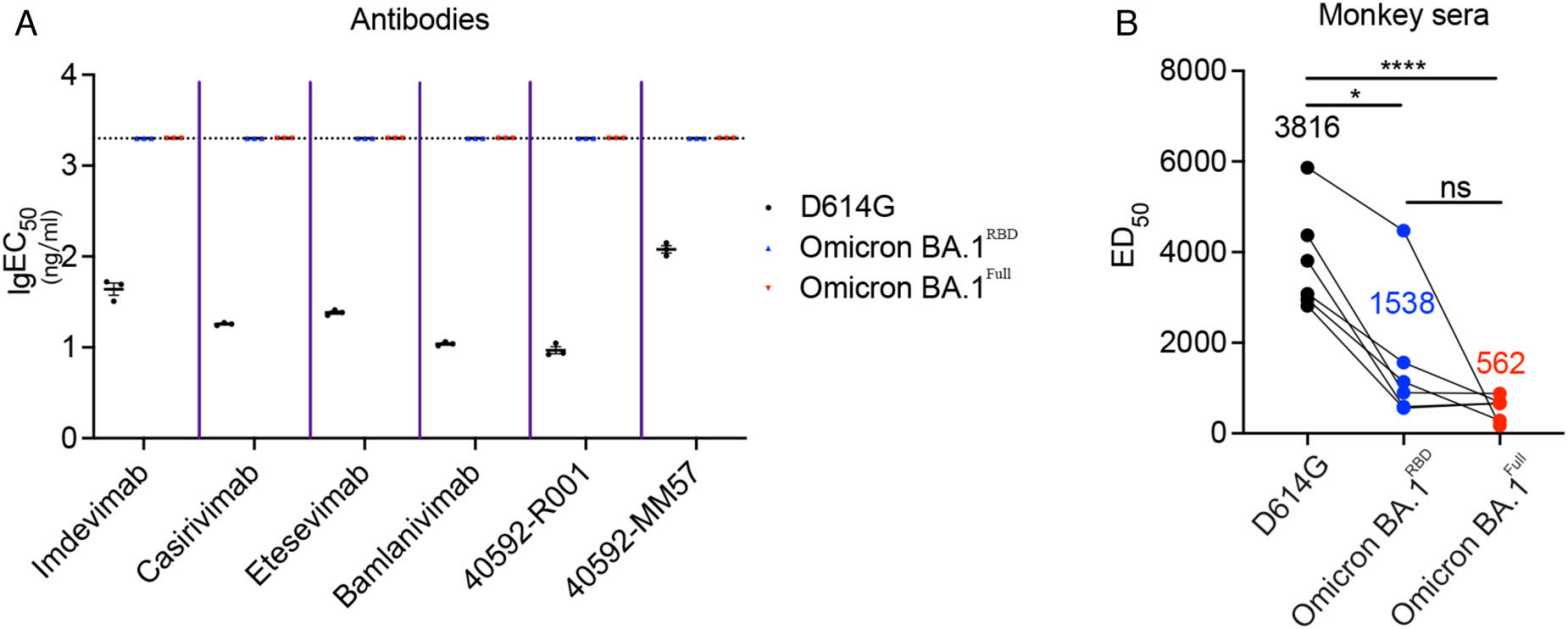
Neutralization activity of monoclonal antibodies and immune sera from recombinant RBD protein vaccinated nonhuman primates against Omicron BA.1 variant pseudotyped viruses. (A) Mean ED_50_ values derived from neutralization assays of six monoclonal antibodies. All of the monoclonal antibodies showed neutralizing activity against pseudotyped viruses containing the D614G variant. Remarkably, neutralizing activity was beyond the limit of detection for any of the Omicron BA.1 pseudotyped viruses. We did not perform statistical analyses on these data. *x*‐axis shows different antibodies, *y*‐axis shows lg(EC_50_) (ng/mL), concentrations for 50% effective neutralization. (B) Mean neutralizing titers from neutralizing assays of immune sera from recombinant RBD protein vaccinated nonhuman primates (n = 6). *x*‐axis shows different SARS‐CoV‐2 variants, *y*‐axis shows ED_50_, the values of serial dilution for 50% effective neutralization. The neutralizing titer against pseudotyped viruses containing the D614G mutation is high. However, the neutralizing titer was significantly reduced for Omicron BA.1^RBD^ (one‐way ANOVA; *p* < 0.05) and Omicron BA.1^Full^ (one‐way ANOVA; *p* < 0.0001)

The Omicron BA.1 variant may escape neutralization by most of the existing monoclonal antibodies.^21^ We, therefore, assessed neutralization activity of several monoclonal antibodies, including four of which are in clinical use which are (Bamlanivimab [LY‐CoV555], Etesevimab [LY‐CoV016], Imdevimab [REGN10987], and Casirivimab [REGN10933], and two SinoBiological SARS‐CoV‐2 spike neutralizing antibodies, 40592‐R001 and 40592‐MM57. RBD antibodies were classified for four main classes (Cao Y et al. Omicron escapes the majority of existing SARS‐CoV‐2 neutralizing antibodies). Casirivimab and Etesevimab belong to class 1, and Bamlanivimab and Imdevimab belong to class 2 and class 3, respectively. 40592‐R001 and 40592‐MM57 are two SARS‐CoV‐2 Spike neutralizing antibodies produced by SinoBiological, which specifically target the RBD of SARS‐CoV‐2 and potently block its binding to the ACE2 receptor on target cells. We determined the neutralizing activity of these antibodies by evaluating the 50% effective dose (ED_50_) of the monoclonal antibodies to reduce pseudovirus infection of HEK‐293T cells stably expressing ACE2 and TMPRSS2. Our results show that pseudoviruses harboring the D614G substitution are sensitive to inhibition by all six monoclonal antibodies (Figure 3A). However, in the presence of Omicron BA.1^Full^ and Omicron BA.1^RBD^ pseudovirus, the ED_50_ for all six monoclonal antibodies were beyond the detection limit of the assay (Figure 3A).

### 2.4 Neutralization activity of immune sera from RBD‐vaccinated nonhuman primate

A number of studies have demonstrated that the Omicron BA.1 variant extensively escapes neutralization mediated by antibodies from mRNA vaccines.^17^, ^20^, ^23^, ^24^ We tested the neutralization activity of sera obtained from nonhuman primates immunized with a recombinant RBD protein vaccine (n = 6). We have reported high anti‐RBD antibody levels and robust neutralization activities against SARS‐CoV‐2 WT and B.1.427/429 variant pseudovirus.^25^ We evaluated the neutralizing activity of serum from vaccinated monkeys using the neutralizing assay outlined above. Our data showed that as compared to pseudovirus containing only the D614G substitution, Omicron BA.1^Full^ and Omicron BA.1^RBD^ pseudovirus reduced the neutralization activity of the immune sera from vaccinated monkeys (Figure 3B). However, it should be highlighted that while the neutralization activity was reduced versus the D614G pseudovirus, monkey sera vaccinated by RBD protein still showed a relatively high titer of neutralizing antibodies against the Omicron BA.1^Full^ and Omicron BA.1^RBD^ pseudovirus, and suggests that a recombinant RBD protein vaccination strategy may confer enhanced protection against the Omicron BA.1 variant.

### 2.5 Neutralization activity of immune sera from vaccine recipients

**FIGURE 4:**
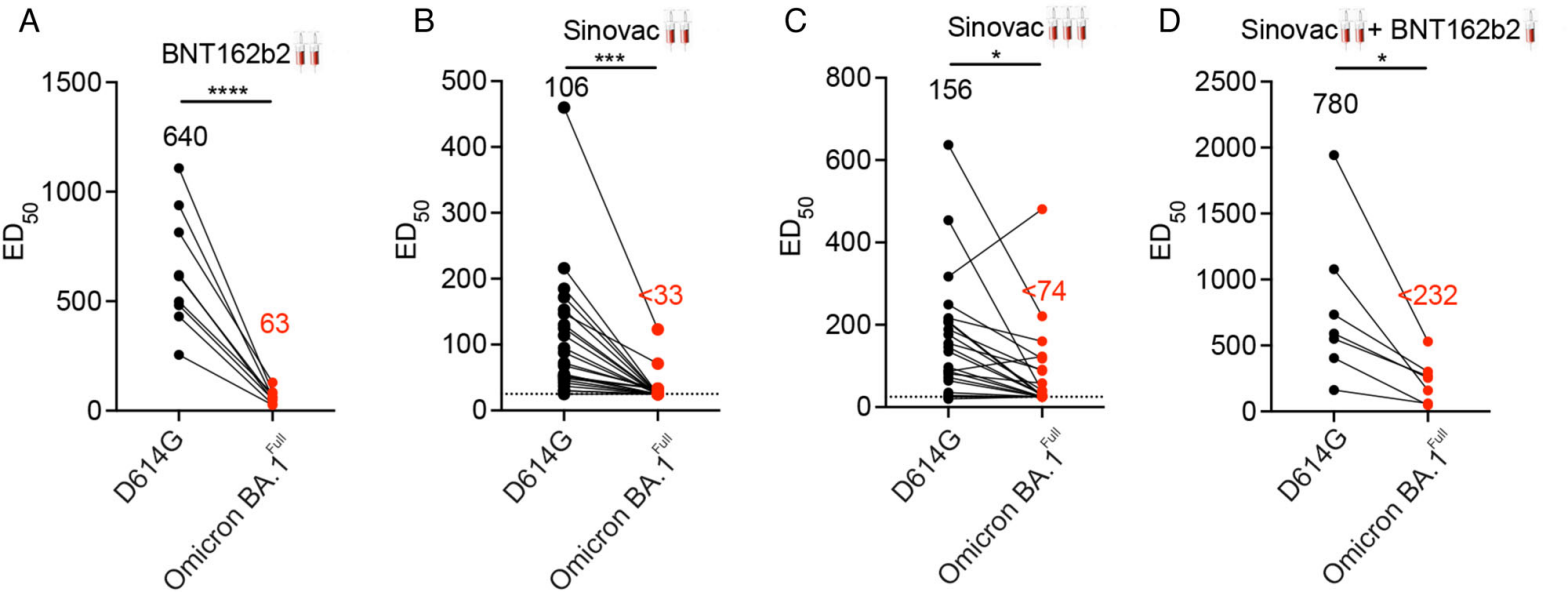
Neutralization activities of immune sera from recipients of standard two‐dose CoronaVac or BNT162b2, or three‐dose vaccination against the Omicron BA.1 variant. Graphs show neutralizing dilutions which yielded 50% neutralizing activity for two‐dose BNT162b2 (n = 9) (A), two‐dose CoronaVac (n = 23) (B), homologous CoronaVac booster (n = 24) (C), and heterologous BNT162b2 booster (n = 7) (D). Consistent with our other findings, the Omicron BA.1 variant showed reduced sensitivity to the neutralizing activity of immune sera in all the different vaccination regimen. Most of the immune sera from recipients of two doses of CoronaVac vaccine and many immune sera from recipients of a booster of CoronaVac vaccine had neutralizing activities against the Omicron BA.1 pseudotyped viruses which was beyond the detection limit

We extended our investigation to evaluate the neutralizing activity of serum from recipients of two doses of either the BNT162b2 or the CoronaVac vaccines. Our analysis revealed a significant reduction in the neutralization activity of the BNT162b2 sera (n = 9) against either the Omicron BA.1^Full^ and Omicron BA.1^RBD^ pseudovirus versus the D614G pseudovirus (Figure 4A). The fold reduction against the D614G was 10.2‐fold for the Omicron BA.1^Full^ and 2.5‐fold for the Omicron BA.1^RBD^. Sera from two‐dose CoronaVac vaccination recipients (n = 10) also showed a similar reduction in the neutralization activity (Figure 4B). The neutralizing titer against Omicron BA.1^RBD^ and Omicron BA.1^Full^ of a number of samples were beyond the detection limit. This suggests that the Omicron BA.1 variant may completely escape neutralizing antibodies in some CoronaVac recipients. These samples were arbitrarily assigned a neutralizing titer of 25 (the minimum fold dilution in these experiments).

Booster vaccinations can increase the level of neutralization antibodies against the Omicron BA.1 variant.^18^, ^26^, ^27^ The choice of homologous or heterologous booster regimens may also influence the extent of protection.^16^ We found sera from recipients of a homologous booster regimen (three doses of the CoronaVac vaccine, n = 24) showed robust neutralization activity against pseudovirus harboring the D614G substitution (Figure 4C). Consistent with our observations above, their neutralization activity against Omicron BA.1^Full^ was 1.9‐fold lower though neutralization activity was detectable in all sera samples. A similar pattern was observed for sera from a heterologous booster regimen (two doses of CoronaVac vaccine followed by one dose of BNT162b2 vaccine, n = 6): the third dose of BNT162b2 elicited stronger neutralizing activity against pseudovirus with the D614G substitution (Figure 4D). They thought the activity was significantly reduced against the Omicron BA.1^Full^. In comparison of the efficacy between homologous and heterologous booster regimens, the latter one showed significantly higher neutralizing activity against D614G and Omicron BA.1^Full^ substitution, though the efficacy of both booster regimens reduced remarkably against Omicron BA.1^full^. There is no difference between the Omicron BA.1^RBD^ and the Omicron BA.1^Full^ in BNT162b2, and two doses CoronaVac or CoronaVac + BNT booster (data not shown).

## 3 DISCUSSION

Our study results are consistent with an emerging body of evidence that mutations in the spike protein of the Omicron BA.1 variant confer increased resistance to neutralizing antibody activity from either monoclonal antibodies or vaccination.^1^, ^3^ In particular, our study revealed that the Omicron BA.1 variant showed little sensitivity to all of the neutralizing antibodies we evaluated in this study, consistent with previous observations.^21^ Emerging cryoelectron microscopy comparisons of the Omicron BA.1 and Delta spike protein structure suggest that the more compact conformation of the Omicron BA.1 spike may contribute to its neutralization by antibodies.^28^ Our study adds to this body of emerging evidence that the resistance to neutralizing antibodies is conferred largely by the mutations in the RBD, since pseudovirus harboring additional spike protein mutations did not significantly change the resistance to neutralizing activity. Homologous or heterologous booster vaccinations can increase the neutralizing activity against the Omicron BA.1 variant, but consistent with other studies, the magnitude of this protection is lower than against the D614G variant.^17^, ^29^ Nevertheless, our results from nonhuman primates show that a recombinant RBD protein vaccine may be a promising approach for future vaccine development.

Our data also showed that Omicron BA.1 pseudovirus has lower infectivity and reduced membrane fusion compared with pseudovirus harboring the D614G substitution, consistent with some observations that cell entry by the Omicron BA.1 variant was impaired.^30^ However, in similar experiments performed in HEK‐293T cells expressing ACE2 but not TMPRSS2, the infectivity of the Omicron BA.1 variant is higher when compared with SARS‐CoV‐2 pseudoviruses harboring the D614G substitution.^31^ Indeed, the Omicron BA.1 variant shows less efficient replication and fusion activity than the Delta variant in TMPRSS2 expressing cells,^32^ which highlights the critical importance of TMPRSS2 for the pathogenicity of the Omicron BA.1 variant. Our study investigated the mutations found in the spike protein only. Other SARS‐CoV‐2 structural proteins, such as the membrane and envelope proteins, and mutations in them, may also impact the pathogenicity of the Omicron BA.1 variant. For instance, the envelope protein of SARS‐CoV‐2 is an ion channel that is involved in the maturation and assembly of new SARS‐CoV‐2 particles.^33^ The Omicron envelope protein harbors an additional T9I substitution.^33^ The impact of this, together with the triple mutations D3G, Q19E, and A63T in the membrane protein on the pathogenicity of the Omicron variant is not known. Furthermore, continued emergence of additional variants with novel mutations, for instance in the BA.2 sublineage of the Omicron variant, may further impact the pathogenicity of SARS‐CoV2, and perhaps the efficacy of neutralizing antibodies.^34^ Adequate control of the COVID‐19 pandemic may be dependent on the development of more efficacious antibodies. The data we present in this study regarding the neutralizing efficacy of a recombinant RBD protein vaccine against the Omicron variant are indeed promising. A number of other recombinant protein vaccines are undergoing clinical trials with promising results.^35^, ^36^, ^37^ However, to move ahead of the COVID‐19 pandemic may require the development of a pan‐coronavirus vaccine.^38^, ^39^

Nevertheless, our findings add to an emerging clinical picture that while the efficacy of neutralizing antibodies is impaired against the Omicron BA.1 variant, the severity of the disease caused by the Omicron BA.1 variant is less compared with the Delta variant. A large comparative analysis in England for the risk of hospitalization and death following infection with the Omicron BA.1 or Delta variant shows a substantially reduced hazard ratio for infection with the Omicron BA.1 variant.^40^ Our results also add to a growing body of evidence regarding immune escape by the Omicron BA.1 variant, and shed light on some of the differences in infectivity and cell–cell fusion conferred by non‐RBD mutations of the spike protein. The enhanced protection offered by recombinant RBD protein vaccines may be an encouraging approach to alter the trajectory of this pandemic in our favor.

## 4 MATERIALS AND METHODS

### 4.1 Cell culture

HEK‐293T cells with (from Creative Diagnostics, USA; CSC‐ACE02) and without (from Procell, China; CL‐00005) stable expression of ACE2 and TMPRSS2 were cultured in complete Dulbecco's Modified Eagle's Medium (DMEM, Thermo Fisher, USA) supplemented with 10% fetal bovine serum (FBS, PAN‐Biotech, Germany), 1% penicillin‐streptomycin (100 U) (Gibco, USA) at 37°C with 5% CO_2_. Stable expressions of ACE2 and TMPRSS2 were achieved by culturing the cells in the presence of puromycin (0.5 μg/mL). Puromycin was removed from the cultures prior to experiments. Cells were subcultured every 3–4 days by digestion with 0.25% trypsin (Gibco).

### 4.2 Omicron BA.1 variant S protein expression plasmids

The Omicron BA.1 variant spike gene with the full complement of mutations (Omicron BA.1^Full^) and that with only mutations in the RBD (Omicron BA.1^RBD^) were synthesized and subcloned into the pCAGGS vector (Genscript, Nanjing, China). The expression plasmid containing the spike gene with the D614G substitution was previously constructed by our group.^25^ All constructs used in this study were confirmed by Sanger sequencing.

### 4.3 Human and nonhuman primate sera

The study protocols for acquiring sera from immunized human subjects and nonhuman primates were approved by the Medical Ethics Committee of the Macau University of Science and Technology. Sera from consented vaccination recipients who have completed two doses of either CoronaVac (Sinovac) (n = 10), BNT162b2 (BioNTech) (n = 9), or two‐dose CoronaVac recipients boosted homologously (n = 12) or heterologously (with BNT162b2) (n = 6) were obtained by phlebotomy. Sera used in the evaluation of vaccination were obtained with an average of 14 days following the second or booster dose. Subjects recruited for the evaluation of booster vaccinations received their vaccinations between 3 and 6 months after the second dose. Sera (n = 6) from recombinant RBD protein vaccinated Cynomolgus macaques (*Macaca fascicularis*) (5–9 years old) were obtained as previously described.^25^

### 4.4 Monoclonal antibodies

The following monoclonal antibodies were used in this study: Casirivimab‐derived recombinant monoclonal mouse IgG2a (srbdc3‐mab10, InvivoGen), Imdevimab‐derived recombinant monoclonal mouse IgG2a (srbdc4‐mab10, InvivoGen), Bamlanivimab‐derived recombinant monoclonal mouse IgG2a (srbdc5‐mab10, InvivoGen), Etesevimab‐derived recombinant monoclonal mouse IgG2a (srbdc6‐mab10, InvivoGen), SARS‐CoV‐2 (2019‐nCoV) Spike Neutralizing Antibodies (Rabbit Mab [40592‐R001, SinoBiological] and Mouse Mab [40592‐MM57, SinoBiological]).

### 4.5 Production and quantification of pseudotyped viruses

HEK‐293T cells were seeded at a density of 5 × 10^6^ cells in a 100 mm dish and were transfected with 12 μg pLOVE‐luciferase‐EGFP plasmid, 6 μg psPAX2, and 2 μg spike protein variant plasmids using Lipofectamine 3000 (Invitrogen) according to the manufacturer's instructions. The media of transfected cells were replaced after 6 to 8 h, and SARS‐CoV‐2 pseudotyped viruses were harvested and filtered through a 0.45 μm filter 48 h after transfection. RNA extracted from pseudotyped viruses (MiniBEST Viral RNA/DNA Extraction Kit Ver.5.0; TaKaRa, 9766) were reverse transcribed using the HiScript III All‐in‐one RT SuperMix Perfect for qPCR (Vazyme, R333‐01). RT‐PCR was performed using the TransLv Lentivirus qPCR Titration Kit (TransGen, FV201).

### 4.6 Infectivity assay

HEK‐293T cells stably expressing ACE2 and TMPRSS2 were seeded at a density of 1 × 10^4^ cells in 96‐well plates. Pseudotyped viruses were diluted to 8 × 10^4^ particles per 100 μL in DMEM medium, and added to each well. Following a media change after 12 h, luciferase activity was assayed after another 48 h. Infected cells were lysed in the presence of luciferase substrate. Luminescence signal was detected using the LumiStation 1800 Luminescence Microplate Reader (Shanghai Flash Spectrum Biotechnology Co., China).

### 4.7 Cell–cell fusion assay

HEK‐293T cells were transfected with pCMV‐EGFP alone or in combination with or pCAGGS‐SARS‐CoV‐2 spike and cultured in DMEM containing 10% FBS at 37°C for 48 h. HEK‐293T cells stably expressing ACE2 TMPRSS2 were seeded at a density of 5 × 10^5^ in 12‐well plates at 37°C for 5 h, following which EGFP and/or SARS‐CoV‐2 spike transfected HEK‐293T cells were added at a density of 1 × 10^5^ cells. After coculture at 37°C for 12 h, fused cells were counted under an inverted fluorescence microscope (Leica, DMIL LED). The following criteria were used for fusion: (1) Fused cells increased in size by at least twofold, and (2) the intensity of fluorescence in the fused cells was reduced. Five fields were randomly selected in each well to count the number of fused and unfused cells under an inverted fluorescence microscope. The relative fusions were calculated by the percentage of fused cells in Omicron BA.1^RBD^ or Omicron BA.1^Full^ divided by the percentage of fused cells in D614G. All experiments were performed at least three times.

### 4.8 Neutralization assay

HEK‐293T cells stably expressing ACE2 and TMPRSS2 were seeded at a density of 1 × 10^4^ in 96‐well plates. Pseudotyped viruses were incubated in the absence or presence of serial dilutions of sera samples or monoclonal antibodies (1:25, 50, 100, 200, 400, 800, 1600, 3200, 6400, 12,800) for 1 h at 37°C before being added to cells. Cells were incubated at 37°C for 12 h, following which the viruses were removed by a media change. The cells were lysed in the presence of luciferase substrate 48 h later, and the luminescence signals were detected using the LumiStation 1800 Luminescence Microplate Reader (Shanghai Flash Spectrum Biotechnology Co., Ltd, China). The sample ED_50_ (median effective dose) was calculated using the Reed–Muench method.^41^

### 4.9 Quantification and statistical analysis

GraphPad Prism 8 was used for plotting and statistical analysis. Summary statistics were expressed as mean ± SEM. Statistical analyses on the influence of different pseudotyped viruses were performed using one‐way ANOVA. Corrections for post‐hoc pairwise comparisons were performed using the Tukey method. *p*‐values of less than 0.05 were considered to be statistically significant.

## CONFLICT OF INTEREST

Kang Zhang is an editorial board member of MedComm. Author Kang Zhang was not involved in the journal's review of, or decisions related to, this manuscript. The other authors declared no conflict of interest.

## ETHICS STATEMENT

Study protocol was approved by the Institutional Ethical Review Committee of Macau University of Science and Technology, the application number is 20210913‐001.

## AUTHOR CONTRIBUTIONS

Z.Z., P.D., N.L., X.X., S.T., Q.D., T.W., M.Y., and Z.L. performed the experiments, collected, and analyzed the data. K.Z. and G.L. conceived this research direction and supervised the project. Z.Z., N.L., and G.L. wrote the manuscript. M.M., D.T.B‐H., O.M., W.S.N., U.M.L., W.H.T., and K.L. provided technical assistance. D.T.B‐H. revised the manuscript. All authors discussed the results and reviewed the manuscript.

## Data Availability

The data included in this study are available upon request from the corresponding author.
